# Estimation of Radionuclide Concentrations and Average Annual Committed Effective Dose due to Ingestion for the Population in the Red River Delta, Vietnam

**DOI:** 10.1007/s00267-018-1007-8

**Published:** 2018-02-16

**Authors:** Tran Thi Van, Luu Tam Bat, Dang Duc Nhan, Nguyen Hao Quang, Bui Duy Cam, Luu Viet Hung

**Affiliations:** 1 Department of Science, Technology, Environment and International Cooperation, Directorate for Roads of Vietnam, D20 Lot, Ton That Thuyet Street, Cau Giay, Hanoi, Vietnam; 2 Research Institute of the Environment and Social Problems, 6 Vu Huu, Thanh Xuan, Hanoi, Vietnam; 3 Viet Nam Atomic Energy Institute, 59 Ly Thuong Kiet, Hanoi, Vietnam; 4 University of Natural Sciences, Hanoi National University, 235 Nguyen Trai, Thanh Xuan, Hanoi, Vietnam; 5 Department of Science and Technology, Ministry of Defense, 15 Hoang Dieu, Ba Dinh, Hanoi, Vietnam

**Keywords:** ^232^Th and ^238^U radioactive chain, Potassium-40, Polonium-210, Annual committed effective dose, Red River Delta, Vietnam

## Abstract

Radioactivity concentrations of nuclides of the ^232^Th and ^238^U radioactive chains and ^40^K, ^90^Sr, ^137^Cs, and ^239+240^Pu were surveyed for raw and cooked food of the population in the Red River delta region, Vietnam, using α-, γ-spectrometry, and liquid scintillation counting techniques. The concentration of ^40^K in the cooked food was the highest compared to those of other radionuclides ranging from (23 ± 5) (rice) to (347 ± 50) Bq kg^−1^ dw (tofu). The ^210^Po concentration in the cooked food ranged from its limit of detection (LOD) of 5 mBq kg^−1^ dw (rice) to (4.0 ± 1.6) Bq kg^−1^ dw (marine bivalves). The concentrations of other nuclides of the ^232^Th and ^238^U chains in the food were low, ranging from LOD of 0.02 Bq kg^−1^ dw to (1.1 ± 0.3) Bq kg^−1^ dw. The activity concentrations of ^90^Sr, ^137^Cs, and ^239+240^Pu in the food were minor compared to that of the natural radionuclides. The average annual committed effective dose to adults in the study region was estimated and it ranged from 0.24 to 0.42 mSv a^−1^ with an average of 0.32 mSv a^−1^, out of which rice, leafy vegetable, and tofu contributed up to 16.2%, 24.4%, and 21.3%, respectively. The committed effective doses to adults due to ingestion of regular diet in the Red River delta region, Vietnam are within the range determined in other countries worldwide. This finding suggests that Vietnamese food is safe for human consumption with respect to radiation exposure.

## Introduction

Natural radioactivity is caused by the presence of naturally occurring radioactive matter (NORM) in the Earth crust. Among others, potassium-40 (^40^K), uranium-238 (^238^U and its decay series), and thorium-232 (^232^Th and its decay series) represent the group of NORM radionuclides. In addition to the NORM, several artificial radionuclides, e.g., strontium-90 (^90^Sr), cesium-137 (^137^Cs), and plutonium-239+240 (^239+240^Pu), which were released due to the human activities during the late 1950s and early 1960s are still present in our environment (Vaca et al. 2001; Cochran et al. 1987). These natural and artificial radionuclides are long lived, the half-lives (T_1/2_) of ^40^K, ^238^U, ^232^Th, ^239^Pu/^240^Pu, ^137^Cs, and ^90^Sr are 1.3E9 a, 4.5E9 a, 1.4E10 a, 24E3/6.56E3 a, 33 a, and 28 a, respectively, and nowadays, these radionuclides are typically present in air, soil, and water in different levels of activity. Natural and artificial radionuclides are also found in terrestrial and aquatic food chains, with subsequent transfer to humans through ingestion of food and also through the inhalation of suspended dust in air.

Food is known to contain natural and artificial radionuclides that, after ingestion, contribute to an effective internal dose. It has been estimated that a large portion, at least one-eighth, of the mean annual dose due to natural sources is caused by the intake of food (Giri et al. 2013). Average radiation doses to various organs of the body also represent an important pathway for long-term health considerations.

The potential harmfulness of radionuclides is based on their long half-lives and chemical behavior. Thorium-232 is mainly radiotoxic; ^238^U is both radiotoxic as well as chemically toxic, whereas ^40^K, ^90^Sr, and ^137^Cs are radiotoxic as well as nutritionally important elements and minerals (Tykva and Sabol 1995). Owing to the health risks associated with the exposure to indoor radiation, many governmental and international bodies such as the International Commission on Radiological Protection (ICRP), the World Health Organization (WHO), International Atomic Energy Agency (IAEA), etc. have adopted strong measures aimed at minimizing such exposures.

In Vietnam, the outdoor and indoor average annual effective dose due to external exposure from the surface soil has been estimated for the population throughout the country, and it was as high as 0.08 and 0.46 mSv a^−1^, respectively, resulting in the total annual external effective dose to be of 0.54 mSv a^−1^ (Ngo et al. 2012). The annual effective dose due to inhalation of radon (^222^Rn) and thoron (^220^Rn), the progenies of the ^238^U and ^232^Th radioactive decay series, respectively, has also been estimated for the population in Hanoi city, the capital of Vietnam, and it was around 1.1 mSv a^−1^ (Dang et al. 2012) that was within the range of the doses found in other countries in Asia (Sathish et al. 2012; Khan et al. 2012). However, the average committed effective dose due to food ingestion was not investigated yet, although several research programs related to the determination of radioactive concentrations of natural and artificial radionuclides in food have been conducted (Nguyen et al. 2009).

The aim of this study was to survey for the concentrations of radionuclides in NORM and artificial radionuclides, chiefly of the ^90^Sr, ^137^Cs, and ^239+240^Pu in raw (not cooked) and ready-to-eat (cooked) food, representing for the major diet of the Vietnamese population to elucidate the effluence of the processing method to the concentration of the radionuclides in food and ultimately to estimate the annual committed effective dose through ingestion.

## Method

### Study Area

**Fig. 1:**
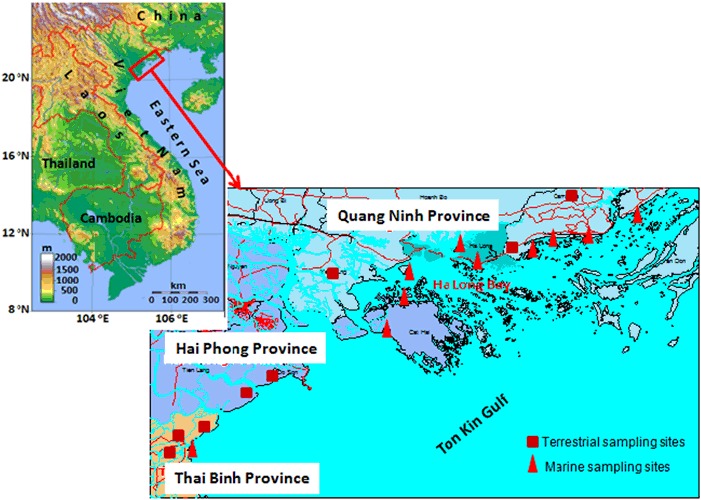
A sketch of the study area and sampling locations

The study was conducted for Thai Binh, Hai Phong, and Quang Ninh, three coastal provinces of the Red River delta, North Vietnam. Figure 1 depicted a map of the study site and the sampling locations.

The coastal provinces were chosen for this study because Thai Binh is the most agricultural productive province with rice production not only for the local consumption, but also for selling to other provinces in North Vietnam. Hai Phong is one of the biggest ports in Vietnam through which annually million tons of food are imported and exported abroad and food quality control for the imported items is the obligatory requirement. Quang Ninh with the famous Ha Long Bay, the UNESO Heritage receives more than a million domestic as well as foreign tourists (QNTO 2016) annually, so that food safety is essential for the local administration.

The soil for agriculture in this region is alluvial that has been built up by the Red River system from the Holocene, which is around 10–15 ka before the present.

### Food Types and Sampling Procedure

The types of food subjected to this study were chosen based on the main diet of the Vietnamese population that was officially reported by the National Institute of Nutrition, Ministry of Healthcare of Vietnam (NIN 2010) and presented in Table 1 for the adults in the Red River delta region.

**Table 1:** The major diet of the Vietnamese population in the Red River delta region (NIN 2010)

Food	Consumption rate, mean (min–max), g per capita per day
Rice	369.1 (357.2–381.1)
Maize	14.7 (12.3–17.1)
Tubers (cassava and sweet potato)	4.9 (3.0–6.9)
Tofu	34.3 (28.5–40.1)
Nut (sesame)	3.2 (2.4–4.1)
Vegetable (leafy)	176.3 (169.3–183.3)
Vegetable (root)	27.9 (22.7–33.1)
Fruit (bananas)	84.0 (72.4–95.6)
Meat (beef and poultry)	108.3 (102.2–114.5)
Egg	18.6 (16.5–20.7)
Fishes (marine and freshwater)	42.0 (36.8–47.3)
Other seafood (marine oyster and clam)	10.7 (8.7–12.7)

Plain rice was bought directly from several farmers in the locality to have 10 kg from each sampling site. Maize, cassava, leafy vegetables (cabbage and colza), and root vegetables (carrot, turnips, and kohlrabi) were taken directly from the farmers’ fields with around 20 kg each. Tofu, sesame, bananas, beef, pork, poultry, egg, and aqua food (fishes: *Scomberomorus maculatus* and *Cyprinus carpio*, clams: *Meretrix meretrix*, and oysters: *Crassostrea gigas*) were bought to the local market at each sampling location by around 5–7 kg each. All the samples were refrigerated with ice in a chest and transported to the laboratory within the sampling day.

### Sample Treatment in the Laboratory

Upon arrival in the laboratory, bananas were immediately removed from the cover as they were already ripened and then subjected to dryness at 80 °C. The maize and cassava were first removed from their husks and skin, washed off, and then corn kernels were separated from the corncobs; cassava tubers were cut into slices with a thickness of 1 cm. The vegetables were taken from the edible part only, washed off, and chopped. The fishes were removed from all the internals and heads; the poultry was removed from its head, and legs were left with edible parts only. All the meats and fishes were chopped into small pieces suited for putting in 3pi-Marinelli box for γ-spectrometry for raw and cooked but dried food. After pretreatment, all the food were divided into two parts, one for direct analysis as raw food, yet another part will be subject to analysis after cooking. For the former part, the food were dried at 80 °C to dryness and then kept in sealed-off PE bags for a longtime storing before the analysis starts. The moisture content in each food after drying was recorded for further correction for the activity concentration on the dry-weight basis (dw). The latter part of food was kept in a refrigerator awaiting for further treatment to get cooked food. As rice was already dry, it was left without any treatment in HDPE bags and kept in a refrigerator at 4 °C.

To have cooked food, the food were taken from the refrigerator and allowed to thaw and strain in a sieve at room temperature; then, poultry and vegetables were boiled; clams and oysters were steamed; tofu, fishes, and beef were mixed with salt and spices and then fried; and sesame or nut was roasted with salt and then pounded to powder following the Vietnamese cooking tradition. After cooking, the bones of the poultry and fishes were removed off and kept with meat only. The boiled vegetables were separated from the liquor and allowed to drip in a sieve. The cooked food was then dried at 80 °C to dryness. The dried cooked food was kept in sealed PE bags and left in a refrigerator for further treatment for the beta- and alpha-activity measurement. For gamma-spectrometry, these dried samples were ready to analyze.

It was expected that the activity concentrations of ^90^Sr and ^239+240^Pu in food would be very low, so to improve the precision of the analysis, an aliquot of both raw and cooked food was ash to enrich the content of the two radionuclides in the samples. For this, around 500 g of the dry-food samples were subjected to ash in an oven at 450 °C for 5 h. The ash was weighed to calculate the volatile content of the sample and then kept in PE bags with sealing off. For ^210^Po determination, the samples were not ash at all because Po is a volatile element at a temperature higher than 100 °C.

### Analytical Procedure

The food was subjected to quantification for alpha-, beta-, and gamma-activity concentrations. The alpha-emitters were ^210^Po and ^239+240^Pu. The beta emitter was ^90^Sr, and the gamma emitters were ^40^K, ^228^Ac, ^208^Tl, ^234^Th, ^210^Pb, ^214^Bi, ^214^Pb, and ^137^Cs. In the gamma-activity analysis, the samples were tightly sealed off in 3pi-Marinelli boxes for at least 3 weeks to attain radiochemical equilibrium that allows to derive the activity concentration of ^232^Th in food from the mean activity concentrations of ^228^Ac, ^208^Tl, and the activity of ^226^Ra from the mean activities of ^214^Bi and ^214^Pb and the activity of ^238^U from the activity of ^234^Th. All the analyses were conducted at the Institute for Nuclear Sciences and Technologies in Hanoi and at the Nuclear Research Institute in Dalat, Vietnam.

For the gamma-spectrometry, a gamma-spectrometer equipped with an HPGe detector of a wide energy range, from 40 keV to 3.0 MeV (Canberra, USA), was used. The energy resolution of the detector was around 1.8–2.0 keV at a peak of 1.33 MeV. Additionally, shielding and electronics of the equipment were specially designed so that it could identify and quantify ^210^Pb and ^234^Th at 46-keV and 63-keV peaks, respectively, that other equipment could not do because of the strong Compton backscattering in the low-energy region. As the radioactivity in the food was expected to be low, the counting time for each type of food was set at least for 24 h to achieve a counting uncertainty of less than ±30%. The ^40^K, ^137^Cs, ^228^Ac, ^208^Tl, ^234^Th, ^214^Bi, and ^214^Pb nuclides were identified and quantified by the peaks of 1.46 MeV, 661 keV, 911 keV, 583 keV, 63 keV, 609 keV, and 352 keV, respectively.

For ^210^Po and ^239+240^Pu (alpha-emitters) analysis, an alpha-spectrometer equipped with a PIPS (passivated ion-implanted planar silicon) detector (α-Analysts, Canberra, USA) with an area of 450 mm^2^ and energy resolution of 18 keV was used. Because the energy of α-particles from the ^239^Pu and ^240^Pu decays is very close to each other, α-decay of ^239^Pu emits energy at 5.15 MeV but that of ^240^Pu does emit at 5.16 MeV, which made the currently available PIPS detectors not be able to separate the two nuclides, hence, in the environmental studies, the activity concentration of the two isotopes is summed up.

For ^90^Sr (a beta emitter) activity determination, a liquid scintillation counter (LSC, HP USA) TRICarb 3770 with ultra-low background was used.

Details of procedures for sample preparation before quantification of β- and α-activity concentration in food were followed by those that were described elsewhere, e.g., in Dang et al. (2014). Briefly, the procedures were as follows.

### For ^90^Sr Determination

A 100-mg portion of stable strontium from a Sr(NO_3_)_2_ solution as an internal standard (IS) was first spiked to an aliquot of 2–3 g of the food ash and then the content was dissolved in 20 ml of HNO_3_ at 8 M (PA grade, Merck). When the solution was not clear, 1–2 drops of H_2_O_2_ (PA grade, Merck) were added and heated in a hot sand plate. After the dissolution was completed, the solution was evaporated to dryness to remove the H_2_O_2_ excess. The residue remaining was dissolved again in 5 ml of HNO_3_ at 8 M. This nitrate solution was then brought onto a Sr-spec, SRW01 cartridge (Eichrome supplier) to separate and purify Sr by cation exchange. Strontium was then eluted from the cartridge by 10 ml of HNO_3_ at 0.05 M and precipitated with NH_4_CO_3_ at 3 M solution. The chemical recovery yield of the separation procedure was determined by weighing the precipitate (SrCO_3_) after drying it at 105 °C for 3 h. Usually, the chemical recovery yield ranged from 88 to 102%. Afterward, 50 mg of stable yttrium (use solution of Y(NO_3_)_3_) were added to the precipitate and then the mixture was dissolved in 5 ml of HNO_3_ at 0.1 M. The solution was left for 3 weeks to attain the ^90^Sr–^90^Y equilibrium. After 3 weeks, yttrium in the solution was separated from strontium by precipitation with 3 ml of NH_4_OH at 1 M. The precipitate was carefully washed off followed by dryness at 105 °C for 3 h and then subjected to conversion into Y_2_O_3_ by calcinations at 500 °C for 2 h. The yttrium oxide obtained was allowed to cool down in a desiccator and was then weighed to determine the radiochemical recovery yield of ^90^Y. Usually, the radiochemical yield was in between 78 and 98%. The yttrium oxide was dissolved in 10 ml of HCl at 0.01 M and the solution was then subjected to counting for ^90^Y activity on the LSC TRICarb 3770 using Cherenkov’s effect.

### For ^239+240^Po Determination

Fifty (50) mBq of ^242^Pu as an internal standard (Amersham supplier) were spiked to 3–5 g of food ash and then the content was dissolved in HNO_3_ at 8 M in the same manner as it was described above for the ^90^Sr determination. Plutonium was separated and purified by ion exchange using anionit Dowex AG1-X8 packed in a glass column *ϕ*10xH50. Plutonium was eluted from the column by 50 ml of HCl at 10 M containing 2.5 g of NH_4_I. The iodide in the solution was then decomposed by a mixture of concentrated HNO_3_ and HClO_4_ (1:1, v:v). The acidic solution was evaporated to dryness on a hot plate. The residue obtained was dissolved in H_2_SO_4_ at 2 M. This solution is ready to prepare a Pu source for counting for its activity. For this, the solution was adjusted to pH 2–3 using NH_4_OH at 1 M solution. All the content was brought into an electrolytic cell made from Teflon equipped with a stainless-steel planchet ϕ22 mm as the anode and the cathode was made from platinum (Pt). The electrolysis was carried out at room temperature with a current of 0.5 A for 2 h. After the electrolysis was completed, the source was removed from the cell and washed first by deionized water followed by absolute alcohol, and then it was heated on a hot plate till it became glow (the source side must be up) to fix the radionuclide on the substrate. The source was allowed to cool down before counting its activity on the Canberra α-spectrometer with the PIPS detector. The radiochemical recovery yield of the procedure was checked with the internal standard ^242^Pu.

### For ^210^Po Determination

Twenty mBq of ^209^Po (Amersham supplier) were spiked to 3–5 g of dryness at 80 °C food and this content was then subjected to wet decomposition with 20 ml of concentrated HNO_3_ (65%, PA, Merck) on a hot sand plate. If the digest was not clear, some drops of H_2_O_2_ were added. The digestion was continued till the solution became clear. The nitrate solution obtained was evaporated to almost dryness followed by dissolution in HCl at 1.0 M. The evaporation–dissolution cycle was repeated three times to completely destroy the H_2_O_2_ excess. Finally, the residue was dissolved in HCl at 1.5 M for self-electrodeposition on silver planchets. The electrolysis was carried out at 70 °C for 3 h. After the electrolysis was completed, the source was removed from the electrolytic cell and washed with deionized water followed by absolute alcohol and left to dry in a desiccator. The radiochemical recovery yield of the procedure was checked with the activity of the internal standard ^209^Po on the Canberra α-spectrometer with the PIPS detector.

The limit of detection (LOD) of procedures for alpha, beta, and gamma emitters was estimated followed by L’Annunziata (1998) as low as 5 mBq kg^−1^ dw, 50 and 20 mBq kg^−1^ dw food, respectively.

A quality-control program for the analytical results was applied by analyzing the IAEA-414 SRM (standard reference material from the International Atomic Energy Agency, Vienna, Austria) for the activities of ^40^K, ^137^Cs, and other gamma emitters of the ^232^Th and ^238^U series in food, by analyzing the NIST-3454 SRM (standard reference material from the National Institute for Standards and Technology, USA) for activity of ^90^Sr in food, and participating in an IAEA intercomparison exercise with the IAEA-385 for ^238^Pu and ^239+240^Pu determination in the Irish sea sediment. The deviation from the certified values for all the radionuclides under the determination of our laboratory was within the acceptable ranges (Nguyen et al. 2004a).

In this study, the analysis was conducted with at least three replicates for each type of food, and the results were then calculated as arithmetic mean with 1σ standard deviation for each sample from each sampling site.

### Estimate the Average Annual Committed Effective Dose due to Ingestion

The average annual committed effective dose due to the intake of ^238^U, ^232^Th, and its radioactive decay progenies, as well as ^137^Cs and ^40^K in food can be evaluated using the following expression (UN SCEAR 2015):

$$E = \mathop {\sum}\limits_i {\left( {{\mathrm{Q}}_i \ast {\mathrm{C}}_{i,r}} \right)} \ast g_r$$

where *i* denotes a food group (rice, meat, vegetable, etc.); Q_*i*_ and C_*i,r*_ denote the consumption rate (kg y^−1^) and activity concentration of radionuclide *r* in the *i* food group (Bq kg^−1^), respectively; and g_*r*_ is the dose conversion factor for ingestion of radionuclide *r* (Sv Bq^−1^). The recommended dose conversion factors g_*r*_ for the radionuclides studied were taken from the BSS 115 (IAEA 1996). The RadToolbox computer code supplied by the National Regulatory Commission of the United States was used to compute the annual committed effective dose for adults in the Red River delta region based on their diet (Table 1) and the activity concentrations of radionuclides were determined (Table 2).

**Table 2:** Radioactivity concentrations (mean ± standard deviation) of natural radionuclides in food constituting the diet of the population in the Red River delta region (Bq kg^−1^ dw)

No.	Food	^40^K		^232^Th		^228^Ac		^226^Ra		^214^Pb		^214^Bi	
		Raw	Cooked	Raw	Cooked	Raw	Cooked	Raw	Cooked	Raw	Cooked	Raw	Cooked
1	Rice	36 ± 12	23 ± 5	<LOD	<LOD	<LOD	<LOD	<LOD	<LOD	<LOD	<LOD	<LOD	<LOD
2	Other food (maize)	93 ± 28	79 ± 14	<LOD	<LOD	<LOD	<LOD	<LOD	<LOD	<LOD	<LOD	<LOD	<LOD
3	Tubers (casava + sweet potato)	90 ± 44	82 ± 10	<LOD	<LOD	0.56 ± 0.18	0.23 ± 0.12	<LOD	<LOD	<LOD	<LOD	<LOD	<LOD
4	Tofu	347 ± 50	347 ± 55	1.31 ± 0.60	0.93 ± 0.22	1.24 ± 0.60	0.98 ± 0.32	0.70 ± 0.32	0.70 ± 0.32	0.57 ± 0.18	0.57 ± 0.18	0.65 ± 0.23	0.65 ± 0.23
5	Nut	139 ± 60	127 ± 43	0.28 ± 0.11	0.20 ± 0.06	<LOD	<LOD	0.21 ± 0.07	<LOD	0.20 ± 0.11	<LOD	0.22 ± 0.12	<LOD
6	Vegetable (leaves)	72 ± 32	67 ± 20	<LOD	<LOD	<LOD	<LOD	<LOD	<LOD	<LOD	<LOD	<LOD	<LOD
7	Vegetable (tubers)	76 ± 27	53 ± 24	<LOD	<LOD	<LOD	<LOD	<LOD	<LOD	<LOD	<LOD	<LOD	<LOD
8	Fruit (bananas)	63 ± 24	NA	<LOD	NA	<LOD	NA	<LOD	NA	<LOD	NA	<LOD	NA
9	Meat	89 ± 27	66 ± 27	<LOD	<LOD	<LOD	<LOD	<LOD	<LOD	<LOD	<LOD	<LOD	<LOD
10	Egg	44 ± 4	38 ± 6	<LOD	<LOD	<LOD	<LOD	<LOD	<LOD	<LOD	<LOD	<LOD	<LOD
11	Fishes	91 ± 24	98 ± 25	0.36 ± 0.18	0.14 ± 0.05	0.41 ± 0.18	0.14 ± 0.09	<LOD	<LOD	<LOD	<LOD	LOD	<LOD
12	Clam + oyster (aqua food)	73 ± 45	67 ± 46	0.64 ± 0.29	0.21 ± 0.14	0.67 ± 0.21	0.21 ± 0.11	0.31 ± 0.13	<LOD	0.31 ± 0.15	<LOD	0.33 ± 0.16	<LOD

^210^Pb		^210^Po	
Raw	Cooked	Raw	Cooked
0.21 ± 0.14	<LOD	2.00 ± 0.56	<LOD
0.62 ± 0.24	0.30 ± 0.13	3.52 ± 1.15	1.07 ± 0.33
0.32 ± 0.12	0.25 ± 0.12	5.26 ± 1.62	1.32 ± 0.57
3.22 ± 1.51	0.43 ± 0.21	5.22 ± 1.52	1.22 ± 0.52
0.67 ± 0.28	0.31 ± 0.18	3.24 ± 1.41	1.42 ± 0.73
0.79 ± 0.31	<LOD	12.9 ± 1.1	<LOD
0.26 ± 0.15	<LOD	22.3 ± 6.71	<LOD
0.36 ± 0.17	NA	0.35 ± 0.15	NA
<LOD	<LOD	<LOD	<LOD
<LOD	<LOD	<LOD	<LOD
0.47 ± 0.15	<LOD	4.80 ± 1.41	<LOD
1.11 ± 0.28	0.60 ± 0.28	33.5 ± 12.3	3.9 ± 1.6

*NA* not applicable, *ND* not determined

*n* = 18, 15, 12, 24, 18, 24, 24, 18, 21, 12, 15, and 57 analyses for rice, maize, cassava/sweet potato, tofu, nut/sesame, leafy vegetable, root vegetable, bananas, meat, egg, fish, and aqua-food, respectively. LOD of γ (^228^Ac, ^212^Pb, ^212^Bi, ^214^Pb, and ^214^Bi) and α (^210^Po) emitters was 20 mBq kg^−1^ dw and 5 mBq kg^−1^ dw, respectively

## Results

Table 3 presents a list of sampling locations along with the type of food that was collected, and Table 2 summarizes the activity concentrations of radionuclides which were quantified with reasonable uncertainties in raw and cooked food. The results in Table 2 are arithmetic mean values (±standard deviation) calculated for each food type from all the sampling sites. Five particular points were revealed in this study. First, the activity concentration of ^90^Sr, ^137^Cs, and ^239+240^Pu in all the study food was lower than its LOD, respectively, of 50, 20, and 5 mBq kg^−1^ dw. Therefore, it could be expected that the artificial radionuclides would have minor contributions to the committed dose to the local population. Second, the activity concentrations of most radionuclides from the ^232^Th and ^238^U decay series were also lower than their LOD, except for ^40^K, ^210^Pb, and ^210^Po in aqua food and vegetables (both leaves and root), and not much deviated between sampling locations. The maximum deviation from the mean value was less than 50% (Table 2). Third, cooking reduced activity concentrations of radionuclides in cooked food, except for ^40^K, compared to those in raw food (Table 2). Fourth, among the studied food, soybean/nut and tofu (made from soybean) have the highest concentration of ^40^K, ^210^Pb, and ^210^Po. To have tofu from soybean, first, the bean was ground with water and this water will extract the protein content. The extract looks like milk. This milk was then boiled to coagulate the protein together and thus it comes to the surface in the boiler as tofu. Farmers could make tofu in any shape by using an appropriate wood mold. Fifth, the activity concentration of ^40^K in soybean and tofu was not much differed from each other under the processing bean to its product; in average, the ^40^K concentration in soybean and tofu was (347 ± 50) Bq kg^−1^ dw for all sampling sites. It should be noted that rice contains the lowest activity concentrations of all the radionuclides studied compared to those in other food.

**Table 3:** Sampling locations and sample types of the present study

No.	Location	Coordinates	Rice	Maize	Cassava	Nut	Soybean/ tofu	Vegetable (leafy)	Vegetable (root)	Fruit (bananas)	Poultry/Beef	Egg
Terrestrial samples												
1	Cam Pha, QN	21°08.46′N, 107°21.52′E,	x	x		x	x	x	x	x	x	
2	Binh Lieu, QN	21°31.21′N, 107°23.54′E	x		x	x	x	x	x	x	x	x
3	Tien Yen, QN	21°19.26′N, 107°25.1′E	x	x		x	x	x	x	x		
4	Thuy Nguyen, HP	21°03.34′N, 106°39.3′E		x	x		x	x	x		x	x
5	Duong Kinh, HP	20°45.06′N, 106°45.16′E	x	x		x	x	x	x	x	x	
6	Tien Hai, TB	20°17.54′N, 106°34.07′E	x	x		x	x	x	x	x	x	x
7	Tien Hai, TB	20°18.01′N, 106°33.59′E			x	x	x	x	x		x	
8	Thai Thuy, TB	20°32.14′N, 106°34.00′E	x		x		x	x	x	x	x	x

**Marine samples**		Fish	Clam	Oyster
1. Tien Hai, TB	20°17.33′N, 106°35.20′E	x	x	x
2. Ca Ba-1, HP	20°51.30′N, 106°58.58′E		x	x
3. Cat Ba-2, HP	20°48.15′N, 106°56.57′E	x		x
4. Tuan Chau, QN	20°55.39′N, 106°59.22′E		x	x
5. Cai Lan, QN	20°58.32′N, 107°05.05′E		x	x
6. Hon Gai, QN	20°55.13′N, 107°07.06′E	x	x	x
7. Ha Tu, QN	20°58.28′N, 107°13.27′E	x	x	x
8. Quang Hanh, QN	20°59.43′N, 107°15.33′E		x	x
9. Cam Pha, QN	20°59.35′N, 107°19.16′E		x	x
10. Van Don, QN	21°02.59′N, 107°21.52′E	x	x	x

*QN* Quang Ninh,* HP* Hai Phong, *TB* Thai Binh

Fish: *Scomberomorus maculatus* and *Cyprinus carpio;* Clam: *Meretrix meretrix*; Oyster: Pacific oyster (*Crassostrea gigas*)

It seems that potassium is concentrated all in the soybean protein and not much dissolved in water, as well as it is not volatile during the processing of soybean and during cooking tofu (Table 2). Apparently, soybean plants need lots of potassium nutrient to grow and produce beans, and the major source of potassium for the plant should be from fertilizers. Following tofu, in cooked food, the activity concentration of ^210^Po was found to be ranged from its LOD of 5 mBq kg^−1^ dw (rice) to (3.9 ± 1.6) Bq kg^−1^ dw of seafood (Table 2).

Table 4 presents an estimate of the average annual committed dose to adults in the Red River delta region. The dose was 0.32 mSv y^−1^ out of which rice, leafy vegetable, and tofu contributed up to 16.2, 21.3, and 24.4%, respectively.

**Table 4:** Estimated annual committed effective doses to adults in the Red River delta region due to ingestion (excluding water and beverage)

Food	Consumption rate, kg capita^−1^ a^−1^	Annual committed effective dose through ingestion, Sv person^−1^ a^−1^	Contribution of each food type to the total committed effective dose, %
Rice	134.7	5.20E−05	16.2
Maize	5.4	1.09E−05	3.4
Cassava	1.8	4.11E−06	1.3
Tofu	12.5	7.83E−05	24.4
Nut	1.2	3.34E−06	1.0
Vegetable (leaves)	64.3	6.85E−05	21.3
Vegetable (tubers)	10.2	6.32E−06	2.0
Fruit (bananas)	30.7	2.45E−05	7.6
Meat	39.5	2.83E−05	8.8
Egg	6.8	5.61E−06	1.7
Fishes	15.3	1.69E−05	5.4
Other aqua-food	3.9	2.21E−05	6.9
Total		3.21E−04	100.0

## Discussion

As mentioned above, the activity concentrations of ^90^Sr, ^137^Cs, and ^239+240^Pu in all kinds of food from all the sampling sites were lower than the LOD of the respective radionuclides. The reason for this could be due to the low-activity inventory of the artificial radionuclides over the territory of Vietnam on one hand and the environmental behavior of these radionuclides on the other hand. The inventory of ^137^Cs and ^239+240^Pu in the Red River delta region was recorded as high as (1048 ± 143) Bq m^−2^ and (26 ± 4) Bq m^−2^, respectively (Nguyen et al. 2004b) that was two times lower than that found in China or South Korea. Lu and Higgitt (2000) have reported an inventory of ^137^Cs in the catchment of the Yangtze Three Gorges (China) to be of (2158 ± 285) Bq m^−2^. The inventory of ^239+240^Pu in soil from several areas of China was reported to be in the range of (55–65) Bq m^−2^ (Sha et al. 1991). In South Korea, Lee et al. (1996) reported an inventory of ^137^Cs in soil to be as high as (1983 ± 929) Bq m^−2^ and that for ^239+240^Pu to be (55 ± 32) Bq m^−2^.

In soil, plutonium and strontium appear to be more soluble compared to cesium and this made the former nuclides to be able to migrate deeper and wider in soil. Lee et al. (1996) investigated the depth distribution of ^239+240^Pu and ^137^Cs at six sites in South Korea and found a slightly increasing trend of the ^239+240^Pu/^137^Cs activity ratio with increasing soil depth. Nguyen Hao Quang et al. (Nguyen et al. 2004b) investigated the ^137^Cs/^90^Sr activity ratio in the surface soil layer (0–20-cm depth) at 20 locations in the Red River delta region and found that this ratio varied within a wide range, from 2 to 28 with a mean of 9.3 and a standard error of 2.2. Moreover, the ^90^Sr inventory was weakly correlated with that of ^137^Cs as well as with soil properties. This suggested that ^90^Sr is more soluble and tends to wash off horizontally by runoff.

The low activity concentrations of radionuclides from the ^232^Th and ^238^U decay series in food could be explained by the low concentrations in the aquatic environment, as well as low transfer factor (TF) from soil to plant of the nuclides. Luu (2014) has studied the TF of uranium and thorium from soil to colza—a leafy vegetable planted on Eutric Fluvisol which is a typical type of soil in the Red River delta and found that the TF for ^232^Th and ^238^U was (0.029 ± 0.001) and (0.017 ± 0.003), respectively. The concentrations of ^232^Th and ^238^U in soil from the Red River delta region were reported to be as high as (38 ± 5) Bq kg^−1^ and (55 ± 6) Bq kg^−1^, respectively (Ngo et al. 2012).

As seen from Table 2 the activity concentration of ^40^K in boiled rice was reduced by 37% compared to that in raw rice, while in other food, the concentration of the radioactive potassium was almost unchanged by cooking (Table 2). Apparently, potassium was partly concentrated in the rice bran, so it was washed off even before boiling. In other food, potassium as a nutrient that likely being a constituent of tissues, was retained in food during cooking. The reduction of ^210^Pb and ^210^Po concentrations in boiled, steamed, or fried food might be due to the volatilization of the minerals during the cooking of food. It was documented that ^210^Po is partly volatiled at a temperature from 100 °C (Martin and Blanchard 1969).

It was clearly observed that between the ^210^Pb and ^210^Po radionuclides as well as their radiodecay progenitor ^226^Ra, there was no radioactive equilibrium. The activity concentrations of ^226^Ra, ^210^Pb, and ^210^Po in raw aqua food (fish, marine clam, and oyster) were, respectively, around the LOD, (1.1 ± 0.3) and (33 ± 12) Bq kg^−1^ dw (Table 2), while these figures should be low like that for ^226^Ra. It was well documented that the main source of ^210^Po in soil and the marine environment is from the in situ decay of the atmospheric ^210^Pb fallout, but not directly from the decay of ^226^Ra in the local soil (Kim et al. 2000; Xu et al. 2010; Persson and Holm 2011; Tran et al. 2016). The ^210^Pb in the atmosphere was a result of the decay of ^222^Rn that emanated from the Earth crust. Because of the difference in the biological behavior, three radionuclides ^226^Ra, ^210^Pb, and ^210^Po in the environment are difficult to attain their radioactive decay equilibrium. Plants and animals preferentially tend to take up ^210^Po compared to its progenitor (^210^Pb) (Stewart et al. 2005; Fowler 2011), for example, the ^210^Po/^210^Pb ratio in marine mollusca much deviated from 1 ranging from 10 to 1000 depending upon the tissues (Heyraud and Cherry 1979). In this study, the ^210^Po/^210^Pb ratio for the whole tissue of clam (*M. meretrix*) was found to be 30.

The average annual committed effective dose (AACED) for adults in the Red River delta region was estimated to be ranged from 0.24 mSv a^−1^ to 0.42 mSv a^−1^ with an average of 0.32 mSv a^−1^, out of which ^40^K, ^210^Pb, and ^210^Po contributed 38, 10, and 35%, respectively (the calculation not shown here), and the remaining part was the contribution from the ^232^Th and ^238^U and its decay progenies. This estimated dose was in the range of doses determined in other countries in Asia as well as worldwide. Iyengar et al. (2004) reported that the annual committed dose due to ingestion to the population in Bangladesh, China, India, Japan, Pakistan, Philippines, Republic of Korea, and Vietnam ranged from 0.20 to 0.34 mSv a^−1^. Reeba et al. (2017) reported that Indian population living in a high-background radiation area in Kerala would be exposed to an AACED of 0.41 mSv a^−1^ due to the ingestion of primordial radionuclides in food. For adult French population, the AAECD was estimated to be between less than 0.20 mSv a^−1^ to more than 2 mSv a^−1^, depending on the seafood consumption rate. For most French people who consume an average of 4.6 kg a^−1^ seafood like the Vietnamese do, the AACED to them was estimated to be 0.33 mSv a^−1^ (Renaud et al. 2015). For the population worldwide, the total annual effective committed dose was assessed to be 0.290 mSv a^−1^, at which 0.006 mSv a^−1^ was from the inhalation of radionuclides of the thorium and uranium series in air (UN SCEAR 2000).

It was reported that the average median daily dietary intakes of ^210^Po and ^210^Pb for the adult world population were as high as up to 160 mBq per day and 110 mBq per day, corresponding to the annual effective dose of 70 μSv a^−1^ and 28 μSv a^−1^, respectively (Persson and Holm 2011). In this study, we determined the daily intake of ^210^Po and ^210^Pb for adults in the Red River delta region, respectively, to be as high as 254 mBq d^−1^ and 126 mBq d^−1^. The higher ^210^Po intake of Vietnamese population as compared to the average intake of the population in other parts of the world might be due to the habit of Vietnamese to consume with a high rate of tofu and seafood which contain a high concentration of radioactive polonium.

## Conclusions

A survey for radioactive concentrations of radionuclides of the ^232^Th and ^238^U decay series together with those of ^40^K and artificial radionuclides ^90^Sr, ^137^Cs, and ^239+240^Pu in raw and cooked food constituting the regular diet of Vietnamese population in the Red River delta region was conducted. It was revealed that the concentration of the artificial radionuclides in Vietnamese food was very low compared to that of natural radionuclides. Cooking much reduced the activity concentration of radionuclides in food due to the evaporation of the minerals from the boiled, steamed, or fried food. Activity concentrations of radioactive potassium (^40^K) and polonium (^210^Po) in food were higher compared to those of other natural radionuclides. Rice, leafy vegetable, and tofu contributed, respectively, up to 16.2, 24.4, and 21.3% to the average annual committed effective dose of 0.32 mSv a^−1^ of the Vietnamese population in the Red River delta region. The annual effective dose found for the population in the study area is within the range of the doses determined in other countries worldwide, implying that Vietnamese food is safe for human consumption with respect to radiation exposure.
